# Social violence among Thai gender role conforming and non-conforming secondary school students: Types, prevalence and correlates

**DOI:** 10.1371/journal.pone.0237707

**Published:** 2020-08-14

**Authors:** Lan Anh Thi Do, Marc Voelker, Churnrurtai Kanchanachitra, Pimpawun Boonmongkon, Timo T. Ojanen, Nattharat Samoh, Thomas E. Guadamuz

**Affiliations:** 1 Institute for Population and Social Research, Mahidol University, Nakhon Pathom, Thailand; 2 Department of Society and Health, Faculty of Social Sciences and Humanities, Mahidol University, Nakhon Pathom, Thailand; 3 Center of Excellence in Research on Gender, Sexuality and Health, Faculty of Social Sciences and Humanities, Mahidol University, Nakhon Pathom, Thailand; 4 Faculty of Learning Sciences and Education, Thammasat University, Pathum Thani, Thailand; University of Sao Paulo Medical School, BRAZIL

## Abstract

**Background:**

Gender role non-conforming behaviors are a significant risk factor for school-related violence. The objective of this study is to describe the types, prevalence and correlates of social violence among Thai secondary school students, with a focus on gender role non-conformity.

**Methods:**

This article uses secondary data from a national study of 2070 secondary school students aged 13–20 years representing Bangkok and all four regions of Thailand. Students were asked about their gender/sexual identity, self-perception of their masculinity or femininity, and experiences of social violence. Correlates of social violence were examined using multivariable logistic regression models.

**Results:**

Prevalence of social violence victimization was high (57%). Most students considered themselves to be as masculine or as feminine as other members of their sex (82.6%), while 9.1% thought that they were less masculine/less feminine, and 8.3% thought they were more masculine/more feminine. Students who considered themselves less masculine or less feminine than others of their sex (AOR = 1.59, 95% CI: 1.13, 2.25) were more likely to experience social violence, compared to students who considered themselves equally masculine/feminine. Students who self-identified as lesbian, gay, bisexual or transgender (LGBT) (AOR = 1.37, 95% CI: 1.01, 1.86) were also more likely to experience social violence, compared to students who did not identify as LGBT. However, students who considered themselves more masculine or feminine than other students of their sex (AOR = 0.62, 95% CI: 0.44, 0.88) were less likely to experience social violence.

**Discussion:**

Students who identified as LGBT, or considered themselves to be less masculine or less feminine than other students of their sex, had higher odds of social violence victimization. Anti-bullying campaigns need to emphasize that perpetrating social violence is not tolerated, and gender-based violence needs to be included in comprehensive sexuality education curricula.

## Introduction

Violence among children in schools is an important health problem worldwide [1, 2], and has been highlighted as a significant risk factor for various psychosocial health conditions among adolescents [3]. Gender has been identified as a significant predictor of experiencing school-related violence in general, and social violence specifically [4, 5]. In Thailand, the prevalence of social victimization among secondary school students is remarkable, especially among LGBT students. According to a joint report by Mahidol University, Plan International Thailand, and UNESCO, 36% of LGBT-identified secondary school students in Thailand have been socially victimized in the past month; in this report, social violence included being gossiped about, having one’s secrets exposed, having rumors spread of oneself, or being excluded from a group or an activity [6]. Other behaviors that have been considered social violence include behaviors like belittling, humiliating, denigrating, scapegoating, threatening, scaring or ridiculing the victim [7]. Social violence is sometimes called psychosocial violence. One study reported that the prevalence of psychosocial violence among school students aged 11–15 years in Brazil was 62.5% [8].

Social violence has several negative consequences on the health and psychosocial well-being of school students. Previous research has shown that victims are at higher risk of social exclusion, depression, self-harm, and even suicide [7]. A study in Hong Kong found that nearly 40% of LGBT students were socially isolated [9]. In Thailand, gender non-conforming male students have been ridiculed, excluded from becoming student council president, and many are afraid of using school toilets or attending overnight school camps [6]. In the same study, LGBT students, both male and female, were more likely than non-LGBT students to report being victims of bullying, to have thoughts of suicide, to inflict harm on themselves, to consume alcohol, and to skip school [6]. In a recent study, Guadamuz et al. found that sexual and gender minority secondary school students were more likely than non-sexual and gender minority students to experience social victimization, and that students who experienced social victimization were more than three times as likely to use illicit drugs [10].

Research on social violence worldwide is limited generally, and in Thailand specifically [11]. Most research has been small-scale studies using convenience samples, which may not be representative of the whole country. Past studies may not have collected data on gender, sexuality, or gender norms in relation to violence and victimization [7, 12]. While previous studies have documented high prevalence of social violence among LGBT students, specific risk and protective factors of social violence have not been examined [6]. Knowing these factors would assist in the development of preventive interventions to decrease the incidence of victimization [13–17].

Gender role non-conforming students, compared to gender role conforming students, are at higher risk of peer victimisation generally and social victimisation in particular. Gender role conformity refers to conformity with how society generally expects a person of a particular sex (as assigned at birth) to express themselves in everyday life (e.g., act, dress, etc.) [18, 19]. This is usually conditioned early in life, particularly in schools. Students who do not conform to gender role stereotypes, for example, are ridiculed, teased and even experience violence from their peers. In a recent study, Cheung et al. found that both social victimization and sexual victimization among male and female secondary Thai students were strongly associated with depression in the past one month. Moreover, gender role non-conforming female students were more likely to be depressed than their male counterparts [20]. In another study, Guadamuz et al. found that illicit drug use was associated with social victimization, and the prevalence of lifetime illicit drug use was significantly higher among gender role non-conforming Thai students than gender role conforming students. The study also concluded that gender role non-conforming Thai students were more likely to use illicit drugs [10]. Therefore, gender role conformity is an important correlate of violence victimization.

The objective of this study is to describe the types, prevalence and correlates of social violence among secondary school students in Thailand, with a focus on gender role conforming and non-conforming behaviors.

## Methods

### Sample

This article draws on data from a national study of bullying among secondary school students in Thailand that had a particular focus on bullying targeting students who are or are perceived to be transgender or same-sex attracted. The study was a research collaboration between Mahidol University, Plan International Thailand, and UNESCO [6]. Detailed methodology of the study has been previously reported [6, 10].

Briefly, a computerized survey was conducted in the capital city of Bangkok and four provinces representing four main regions of Thailand (Central, Northeastern, Northern and Southern). In each province, three secondary schools were randomly selected, representing one public and one private school in the provincial capital city (or inner Bangkok), and one public school in a district outside the provincial capital (or in suburban Bangkok). Multistage cluster sampling was used to randomly choose one district outside the provincial capital and in suburban Bangkok. Random sampling was then applied to select the schools within each district. The final sample included fifteen secondary schools and 2070 students from grades 7–12 with age range of 13 to 18 years. Of these, 347 students were from Bangkok, 442 students were from Central Thailand, 431 students were from Northern Thailand, 420 students were from Northeastern Thailand, and 430 students were from Southern Thailand. Data collection took place between August and September 2013.

Permission to use this dataset was granted by the Center of Excellence in Research on Gender, Sexuality and Health of the Faculty of Social Sciences and Humanities at Mahidol University. The main study and the subsequent analysis of secondary data were reviewed and approved by Mahidol University Institutional Review Board.

### Measures

The questionnaire used for this research was informed by qualitative research to understand the social and cultural contexts of bullying and gender fluidity among students in Thailand. This included systematic observation, focus group discussions and in-depth interviews with students, school administrators and teachers in both public and private secondary schools in all four regions of Thailand and Bangkok. The questionnaire was then pilot tested to ensure that it was suitable and easy to complete by secondary school students. The computerized self-administered survey was conducted and completed generally within 15–30 minutes. Moreover, the survey questions and related survey measures were reviewed by key informants and stakeholders via a technical review board that included UN representatives, LGBT-identified activists, and members from non-governmental organizations working with Thai students and adolescents.

#### Masculinity/Femininity status

To assess gender role conforming and non-conforming behaviors, we used two questions, one for each sex (assigned at birth). Male students were asked about their perceptions of themselves to assess how “manly” (less/equal/more manly) they are, compared to other male students. Similarly, female students were asked to assess how “womanly” (less/equal/more womanly) they are, compared to other female students. Then, we created a categorical variable by combining these two variables into one variable with three values (less masculine/feminine, equally masculine/feminine, and more masculine/feminine).

#### Gender/Sexual identity

The term “gender/sexual identity” corresponds to colloquial use of the Thai word *phet*, when used for self-identification; the Thai *phet* as used in everyday conversation are categorical combinations of sex assigned at birth, gender identity and sexual orientation [19]. Gender/sexual identity corresponds to gender identity, defined as “a person’s intrinsic sense of being male (a boy or a man), female (a girl or a woman), or an alternative gender (e.g., boygirl, girlboy, transgender, genderqueer, eunuch),” [18] with the proviso that it also incorporates sexual orientation, in other words a sexual preference for members of a particular sex (or more than one sex). In this study, those who identified as *chai* (indicating a “heterosexual cisgender man”) or *ying* (indicating a “heterosexual cisgender woman”) were categorized as non-LGBT, and those who identified with any other response option were categorized as LGBT. In other words, all participants who indicated a gender/sexual identity that differs from the cisgender, heterosexual social norm, were categorized as LGBT.

#### Gender role conforming and non-conforming behaviors

Gender role conforming includes all those who stated “man” or “woman” as their preferred identity term, and stated that they were equally masculine/ feminine as other members of their sex. Gender role non-conforming includes all participants who either identified themselves using any other gender/sexuality term or indicated that they were less masculine/feminine than other members of their sex.

#### Social violence victimization

Social violence victimization covers certain types of violence victimization that Thai students experience [6]. Victims were categorized as having experienced social violence if they indicated victimization in any of the following three questions: (1) having experienced being gossiped, had secrets exposed, had rumors spread; (2) given insulting or mean looks; and (3) being banned and excluded from a group or activity in the past month. The response options referred to the frequency of being victimized as “not once,” “one to three times per month,” “once a week,” and “more than once a week”; students who chose “not once” as their response to all three questions were categorized as not having experienced social violence [10].

### Statistical analysis

Multivariable logistic regression was used to identify correlates of social violence. A binary dependent variable representing any social violence (0 = No, 1 = Yes) was constructed based on students’ statements regarding their experience of three different forms of social violence; this dependent variable took the value 1 if a participant had experienced any social violence in the past month. Independent variables included province, district (rural/urban), age, parents’ highest education, self-assessed level of masculinity/femininity compared to other members of the same sex (less/equally/more masculine/feminine), self-described gender/sexual identity, grade point average (GPA), access to the Internet on a mobile phone/computer, having a steady/casual partner in the past year, alcohol consumption, and substance use (e.g. cannabis, amphetamine pills, crystal methamphetamine, ecstasy/MDMA, sleeping pills/benzodiazepines or injection drug use).

Two multivariable logistic regression models were created: Model 1 included students’ self-assessed masculinity/femininity and other factors and model 2 included gender/sexual identity and other factors. Both models were adjusted for age, GPA, parents’ highest education, province and district. STATA statistical program (Version 14.0, StataCorp, College Station, Texas) was used to perform all statistical analyses.

## Results

Table 1 shows characteristics of the students in the sample, which were included as independent variables in the regression analysis. The geographical distribution of students among the four regions and Bangkok is approximately equal at around 20 percent, except for Bangkok, which only represents 16.8 percent of the total sample. Just over half of participants were from a provincial capital or inner-city district of Bangkok (52.6 percent) and were in the age bracket of 13–15 years (52.5 percent). Most students had a GPA greater than 2.5 (67.4%), nearly all had access to the Internet on a mobile phone or computer (94.9%) and just over half had a steady or casual partner in the past year (55.6%). About one in five students had consumed alcohol (21.3%) and very few had used illicit substances (4.8%). The distribution of gender/sexual identity is that over half of all students identified as *ying* (cisgender heterosexual females) (50.4%) and 37.7% identified as *chai* (cisgender heterosexual males). Among students assigned as female at birth, 7.6% were categorized as LGBT, and among students assigned male at birth, 4.3% were categorized as LGBT. Most students considered themselves to be as masculine or as feminine as other members of their sex (82.6%), while 9.1% thought that they were less masculine or less feminine than other members of their sex, and 8.3% thought they were more masculine or more feminine than other members of their sex.

**Table 1:** Participant characteristics (N = 2070).

Characteristics	N	%
**Province**		
Northeast	420	20.3
Capital	347	16.8
East	442	21.3
North	431	20.8
South	430	20.8
**District**		
Peripheral district	982	47.4
Provincial capital/inner Bangkok district	1088	52.6
**Age (n = 2069)**		
13–15	1087	52.5
16–17	702	33.9
18–20	280	13.6
**Gender/sexual identity**		
Cisgender heterosexual woman (non-LGBT)	1043	50.4
Cisgender heterosexual man (non-LGBT)	781	37.7
LGBT (female sex assigned at birth)	158	7.6
LGBT (male sex assigned at birth)	88	4.3
**Masculinity/Femininity**		
Just as masculine/feminine as boys/girls in general	1710	82.6
Less masculine/feminine than boys/girls in general	189	9.1
More masculine/feminine than boys/girls in general	171	8.3
**GPA**		
GPA< = 2.5	674	32.6
GPA>2.5	1396	67.4
**Parents’ highest education (n = 1869)**		
Primary school or lower	638	34.1
Secondary school/vocational school	768	41.1
Higher than secondary school/vocational school	463	24.8
**Internet use on a mobile phone or on a computer**		
No	106	5.1
Yes	1964	94.9
**Having a steady partner (faen) or a casual partner (kik) in the past 1 year**		
No	920	44.4
Yes	1150	55.6
**Drinking alcohol (n = 2050)**		
No	1613	78.7
Yes	437	21.3
**Substance use**^a^ **(n = 2050)**		
No	1952	95.2
Yes	98	4.8

^a^Substance use includes cannabis, amphetamine pills, crystal methamphetamine, ecstasy/ MDMA, sleeping pills/benzodiazepines or injection drug use.

Table 2 shows the prevalence and types of social violence. More than half of all students (57%) reported that they had experienced at least one form of social violence at their school in the past month. In particular, 46.5% of students reported that they experienced being gossiped about or had rumors spread about them in order to make them look bad to their friends. Moreover, over one third of students (33.5%) thought that they received nasty and disrespectful looks. A lower proportion of students (15.4%) expressed that they were “banned,” in other words, excluded from peer groups or peer activities at their school.

**Table 2:** Frequency and types of of social violence (N = 2070).

Types of school violence	n	%
**Social violence victimization**	**1181**	**57.0**
Gossiped about, had secrets exposed, had rumors spread about oneself	962	46.5
Given insulting or mean looks	693	33.5
Being banned, excluded from groups or activities	319	15.4

### Correlates of social violence

Table 3 shows the results of logistic regression models of social violence, adjusted for age, GPA, parents’ highest education, province and district. In Model 1, students who considered themselves less masculine or less feminine than other students (AOR = 1.59, 95% CI: 1.13, 2.25), students who drank alcohol (AOR = 1.44, 95% CI: 1.12, 1.84), used illicit substances (AOR = 2.88, 95% CI: 1.66, 5.01), had a steady or casual partner in the past year (AOR = 1.49, 95% CI: 1.22, 1.81), or used Internet on a mobile phone or computer (AOR = 1.79, 95% CI: 1.14, 2.81) were more likely to have experienced at least one form of social violence. However, students who considered themselves more masculine or feminine than other students (AOR = 0.62, 95% CI: 0.44, 0.88) were less likely to have experienced at least one form of social violence. In Model 2, students who were categorized as LGBT (AOR = 1.37, 95% CI: 1.01, 1.86), drank alcohol (AOR = 1.42, 95% CI: 1.11, 1.82), used illicit substances (AOR = 2.79, 95% CI: 1.61, 4.85), had a steady or casual partner in the past year (AOR = 1.44, 95% CI: 1.19, 1.76), or used the Internet on a mobile phone or computer (AOR = 1.75, 95% CI: 1.12, 2.75) were more likely to have experienced at least one form of social violence.

**Table 3:** Factors associated with social violence (N = 1848).

	Model 1^a^			Model 2^a^		
Factors	AOR	95% CI		AOR	95% CI	
**Masculinity/ Femininity**						
Less masculine/feminine than others	**1.59**	**1.13**	**2.25**			
Equally masculine/feminine as others	**1**					
More masculine/feminine than others	**0.62**	**0.44**	**0.88**			
**LGBT identity**						
Non-LGBT identity				**1**		
LGBT identity				**1.37**	**1.01**	**1.86**
**Drinking alcohol**						
No	**1**			**1**		
Yes	**1.44**	**1.12**	**1.84**	**1.42**	**1.11**	**1.82**
**Substance use**						
No	**1**			**1**		
Yes	**2.88**	**1.66**	**5.01**	**2.79**	**1.61**	**4.85**
**Having a steady or casual partner in the past 1 year**						
No	**1**			**1**		
Yes	**1.49**	**1.22**	**1.81**	**1.44**	**1.19**	**1.76**
**Internet use on a mobile phone or computer**						
No	**1**			**1**		
Yes	**1.79**	**1.14**	**2.81**	**1.75**	**1.12**	**2.75**

^a^For both models 1 and 2, age, GPA, parents’ highest education, province and district were adjusted.

## Discussion

Our findings indicated that over half of students in Thailand experience social violence (57%). Similarly, Horton found that 57% of Vietnamese lower secondary school students (grade sixth to ninth) reported being bullied in Haiphong city, Viet Nam [21]. Another survey in five Asian countries, including Cambodia, Indonesia, Nepal, Pakistan and Viet Nam found that school-related violence in general was common among secondary school students [22], similar to the findings of UNESCO’s systematic reviews in the Asia-Pacific region [7, 23]. However, these studies did not report on social violence, which refers to specific types of school-related violence. Our study is among the first to report prevalence of social violence.

We also presented correlates of social violence, which were similar with previous general school-related violence studies [6, 22]. However, previous studies did not focus on gender role non-conforming behaviors or LGBT identities. This study is the first to highlight correlates of social violence among Thai secondary school students, including gender/sexual identity and self-assessed level of masculinity/femininity. For example, we found that students who had LGBT identities, or students who considered themselves to be less masculine or less feminine than other students, had higher odds of social violence victimization. This may be attributed to conditioned gender norms and gender discrimination among students [24].

In addition, other correlates also revealed some interesting patterns that confirmed findings from previous studies. These included alcohol consumption [25] and using illicit substances in the past one month [10, 16]. Since alcohol and drugs are often used in peer groups, being in a situation where these substances are consumed might put students at risk by lowering inhibitions among their peers, which may make acting out forms of social violence more likely [1, 10, 13, 15, 22]. We also found that having a steady or casual partner in the past one year was a risk factor for social violence. One reason for this may be that students who have a partner could be seen as breaking Thai social and cultural norms which stigmatize intimate relationships among students as a sign of not being a responsible student. Such stigma could expose them to more violence, not just from their peers but also from their teachers, parents, and intimate partners [17, 26, 27]. Having an intimate partner may also increase the risk of social violence because it can result in sexual/romantic jealousy and competition for partners [12, 28]. Moreover, previous studies have indicated that people with lower socio-economic status are more likely to experience violence [22, 26]. Having a romantic or sexual partner might also be a proxy for a lower socio-economic status, because such relationships are more tolerated in working-class families than in middle or upper class families in Thai society [29, 30].

Moreover, this study found that students who were accessing the Internet on their mobile phones or on computers had higher risks of social violence; conversely, students who did not access the Internet at all were at lower risks. These results are in line with previous studies [22], which found increased Internet access to be significantly associated with experiencing school-related violence. Possible mechanisms include influence of violent videos and images online, as well as access to social media as an additional context in which social violence may take place. Ojanen and colleagues have pointed out that online harassment interlinks with offline violence [12, 31]. Hence, using the Internet involves the risk of facing online harassment, which is associated with an increased risk of being victimized offline as well. However, since only 5.1% of participants were not accessing the Internet, curbing social violence among students by restricting their Internet access may not be feasible.

There were limitations in the study. First, we recognize there may be potential for information bias in self-reporting experiences of social violence, sexual/gender identity, self-perceived masculinity/femininity, having a romantic or sexual partner, alcohol consumption (possibly while being under the legal drinking age) and use of illegal drugs. However, data was collected using a computerized, self-administered survey without school teachers or school administrators present, thus minimizing this bias [12]. Second, the sample was limited to Thai secondary school students; the results may not be generalizable to out-of-school adolescents. Moreover, the three questionnaire items which were used to measure social violence victimization in this study have not been formally validated. However, the original study included a qualitative pilot test phase to ensure that the items included in the survey were culturally relevant to the Thai context and understandable to adolescents.

Despite these limitations, this study is one of the first to assess the relationship between social violence on one hand and gender role conformity and LGBT identities on the other. Interventions should target LGBT-identified students and students who consider themselves less masculine/less feminine than other members of their sex, since they may be targets of social violence among their peers [32]. It seems that Thai students may also not be aware that social violence is a form of violence and should be taken seriously [6]. Schools need to talk about school-related violence that goes beyond physical and verbal bullying. Anti-bullying campaigns to promote an understanding of the types of social violence may be needed with a clear message that perpetrating social violence is not tolerated. Moreover, topics on gender and gender-based violence need to be included in comprehensive sexuality education curricula. Students need to be tolerant of other students’ LGBT identities and expression, as well as tolerant of gender role non-conforming behaviors of other students. Moreover, online and offline networks to assist vulnerable groups and making counseling available at school can help to alleviate some health risks that are correlated with violence victimization, such as alcohol or drug consumption. Finally, interventions should focus on creating a safe environment at school, establishing networks of good peers who can intervene and provide assistance to victims when they witness violence [22].
